# Human Postural Control

**DOI:** 10.3389/fnins.2018.00171

**Published:** 2018-03-20

**Authors:** Yury Ivanenko, Victor S. Gurfinkel

**Affiliations:** ^1^Laboratory of Neuromotor Physiology, IRCCS Fondazione Santa Lucia, Rome, Italy; ^2^Biomedical Engineering Department, Oregon Health and Science University, Portland, OR, United States

**Keywords:** posture control, equilibrium, muscle tone, postural reflexes, multisensory interactions, postural body scheme

## Abstract

From ancient Greece to nowadays, research on posture control was guided and shaped by many concepts. Equilibrium control is often considered part of postural control. However, two different levels have become increasingly apparent in the postural control system, one level sets a distribution of tonic muscle activity (“posture”) and the other is assigned to compensate for internal or external perturbations (“equilibrium”). While the two levels are inherently interrelated, both neurophysiological and functional considerations point toward distinct neuromuscular underpinnings. Disturbances of muscle tone may in turn affect movement performance. The unique structure, specialization and properties of skeletal muscles should also be taken into account for understanding important peripheral contributors to postural regulation. Here, we will consider the neuromechanical basis of habitual posture and various concepts that were rather influential in many experimental studies and mathematical models of human posture control.

## Introduction

Life evolved in the presence of gravity and it has long been recognized, from ancient Greece to our days, that posture is maintained by tonic muscle contractions acting against gravity and stabilizing the positions of body segments. The Greek physician Galen of Pergamon was, probably, the first to introduce the concept of muscle tone in his work “De motu musculorum” (Galen, 1549). From clinical observations, it has long been known that lesions of the central nervous system may result in pronounced changes in posture. Systematic experimental studies of the physiological mechanisms of postural regulation only began a century ago by Sherrington (1906, 1915) and were further developed by Magnus (Magnus and de Klein, 1912; Magnus, 1924) and Rademaker (1931). Various biomechanical and neurophysiological approaches have been used for understanding the mechanisms of balance control (Horak and Macpherson, 1995).

**Figure 1:**
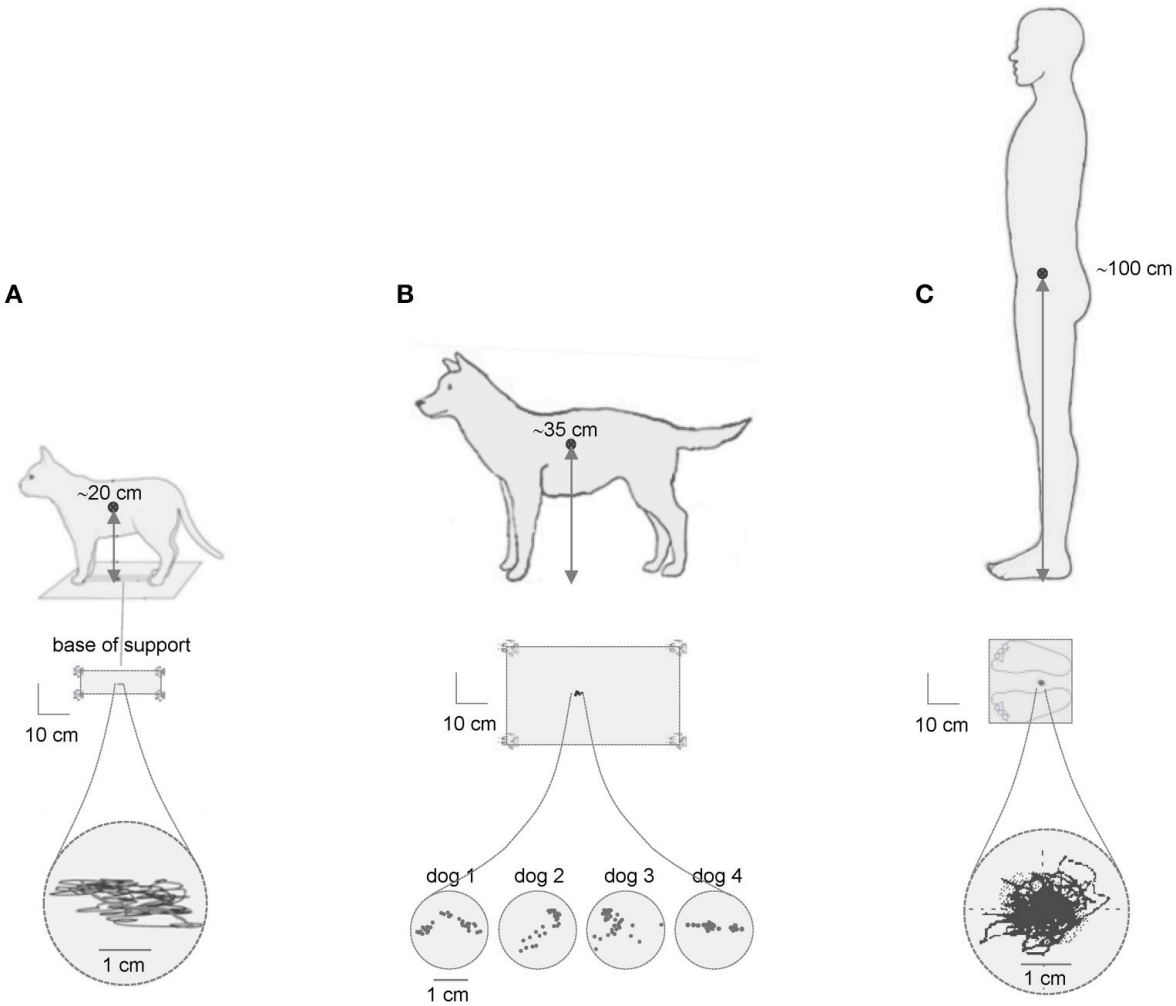
Center of pressure (CoP) fluctuations during quiet standing in the cat **(A)**, dog **(B)** and human **(C)**. Examples of the CoP traces (lower) are adapted from MacPherson and Horak (2012) with permission in **(A)**, redrawn from Brookhart et al. (1965) in **(B)** and modified from Ivanenko et al. (1999) in **(C)**. The size of the base of support is schematically depicted in the middle panels. Note comparable CoP oscillations (~2 cm) in quadrupeds with regard to human despite the 5-fold difference in the height of the center of body mass over the support.

We start this review with an influential scheme of the upright posture control based on the idea of the inverted pendulum and the presence of center of pressure (CoP) oscillations, as important measure of postural stability. In the simplified inverted pendulum model of the upright human posture, the center of body mass (CoM) is the single controlled variable (Winter et al., 2003). In quiet standing, CoP oscillates either side of CoM to keep it in a fairly constant position between the two feet (Figure 1C). Since the center of body mass (CoM) is located relatively high (in the trunk, ~1 m above the ankles that determines the length of the inverted pendulum) and the base of support is relatively small, the posture is inherently unstable. Accordingly, one might conclude that the higher the CoM location, the larger the CoP oscillations. However, this statement is a simplification and appears to be misleading. For instance, Figure 1 illustrates typical examples of the center of pressure fluctuations during quiet standing in the cat, dog and human. Note the similar CoP oscillations (~1–2 cm) despite substantial differences in the height of the center of body mass over the support. Comparable (~1 cm CoP) body sway was also observed in horses (Clayton and Nauwelaerts, 2014) and in rats (~2 cm CoP) trained to stand bipedally (Sato et al., 2015). Therefore, the simple scheme “the lower the CoM, the smaller the CoP oscillations” is deceptive, or at least it cannot be generalized to animals of different size. In addition, the amplitude of CoP oscillations is much smaller than the actual base of support (schematically depicted in Figure 1, middle panels) and would likely provide stability even if it were larger.

Thus, it is important to stress that simple biomechanical considerations can explain the postural behavior only to some extent. Furthermore, the CoP oscillations reflect only an operative level of posture control related to stabilization of specific postural body segments' orientations. However, what are the principles determining habitual postural configurations and tonic muscle activity along the body axis? Postural tone (often associated with antigravity support) represents the tonic activation of muscles in order to provide specific postural attitude and generate force against the ground to keep the limbs extended. The habitual stance vary across animals and may include extended limbs or semiflexed posture. Antigravity support in humans is partly provided by passive bone-on-bone forces in joints, stretched ligaments and muscles, but it also requires active contraction in lower limb, trunk, and neck extensors. The control of postural tone is not simple and requires specialized neural circuitry. Detailed information is required about underlying neural circuitry, as well as about underlying cellular processes in generating prolonged muscle force and stiffness. It is worth noting that postural attitude in different individuals is determined by both individual morphology and specific low-level muscle activity, which can be significantly affected also by different pathological conditions. Integration of several sensory and motor areas has developed through millions of years of life evolution with the purpose of providing accurate regulation of body orientation in the gravity field.

Here we review the experimental challenges that affect the way we define and consider the mechanisms of muscle tone and postural regulation. In the first section, we briefly discuss structural and functional complexity of postural muscles because any reflection on muscle tone and its control should consider the knowledge of the unique structure and properties of skeletal muscles. In the following sections, we discuss the ideas and approaches that represent or represented important conceptual frameworks for investigating human posture control.

## Structural and functional complexity of postural skeletal muscles

The structure and function of skeletal muscle allow wide range of activities, from rapid production of forces and movement to long-lasting maintenance of body segment orientation relative to gravity. In addition, task-specific activation of functionally different types of muscle fibers that compose a given muscle can accomplish a rich repertoire of muscle contractions and energetics of force production. Postural tone is commonly viewed as low-level muscle tension observed in both distal and proximal (trunk and neck) skeletal muscles. Nevertheless, one cannot reflect upon postural tone by considering only the neural input from the sub-cortical and cortical structures. Recent biochemical and biomechanical findings have forced a serious re-evaluation of structural and functional muscle complexity (Knight, 2016). In particular, the sliding filament theory for muscle contraction has been expanded to include regulatory and cytoskeletal proteins that are responsible for the viscoelastic properties of muscle and economy of force production—the key peripheral contributions to postural regulation.

The sliding filament theory is based on the model, where actin and myosin filaments slide past each other, and it was introduced in 1954 independently by the two groups (Huxley and Hanson, 1954; Huxley and Niedergerke, 1954). Hugh Huxley formally proposed the mechanism for sliding filament that is called cross-bridge model. According to his model, filament sliding occurs by cyclic attachment and detachment of myosin on actin filaments. Contraction occurs when the myosin pulls the actin filament toward the center of the A band, detaches from actin and creates a force (stroke) to bind to the next actin molecule. However, the modern views on the mechanism of muscle contraction include three sliding filaments, namely, actin, myosin and titin (Knight, 2016). It is important to note that, in addition to the links to the sarcolemma via T-tubules and the sarcoplasmic reticulum, sarcomeres are linked by other extra-sarcomeric cytoskeletal structures at the Z-disk and M-band. This structure undergoes reversible axial and transverse conformational changes in the contracting sarcomere. The cytoskeletal sarcomeric structure plays a key role in the sliding filament theory (Gautel and Djinović-Carugo, 2016).

In the context of postural function of skeletal muscles and stabilization of body segments, elastic properties of the skeletal musculature and muscle tension are tightly related to regulatory and cytoskeletal proteins. Even though postural muscular activity is rather small, it is worth stressing that any posture is not passive and specific small activity of neck, trunk and limb muscles determines resting tension, axial tone, individual postural attitudes, facial expression, etc. (Jankovic, 2003; Gurfinkel et al., 2006; Wright et al., 2007; Caneiro et al., 2010). Long-lasting maintenance of postural muscle activity (minutes or even hours) is associated with low energy cost. Postural activity normally engages slow muscle fibers, which are more resistant to fatigue. How to control this machinery during posture and small movements that are often present during posture maintenance? In addition to the selective activation of appropriate muscle fibers, a poorly understood, but intriguing, aspect of postural muscle tone encompasses the mechanisms of muscle elasticity, force enhancement and energy conservation.

For instance, static stiffness relies on calcium-dependent stiffening of the activated fibers, independent of crossbridge formation, and titin appears to have all the characteristics required to account for the static stiffness properties (Colombini et al., 2016). Force enhancement may also result from an interaction between an elastic element in muscle sarcomeres and the cross-bridges, which, in turn, interact with the elastic elements to regulate their length and stiffness. A muscle model based on the winding filament hypothesis can predict residual force augmentation in muscles (Nishikawa, 2016). The giant protein nebulin is one of the important regulatory proteins and was proposed to function as a “molecular ruler” to specify the lengths of the thin filaments, which plays a role in numerous cellular processes including regulation of muscle contraction, viscoelastic properties, Z-disc formation, and myofibril assembly (Chu et al., 2016). Interaction between titin and nebulin is still uncertain. Finally, use-dependent changes in muscle fiber composition (Hoppeler, 2016) and progressive decreases in muscle contraction time during child development along with maturation of the central nervous system in the control of posture and movement (Dayanidhi et al., 2013) reflect the functional benefits of such continual maturation and point toward the important role of muscle phenotypic plasticity. The abovementioned topics were traditionally overlooked although the progress in elucidating the molecular mechanisms of muscle contraction opens new avenues in understanding important peripheral contributors to postural regulation and muscle plasticity.

## Conceptual frameworks and approaches for investigating postural control

Upright bipedal stance is traditionally described to depend on sensory (vision, vestibular, and somatosensory) input to provide postural equilibrium and a proper alignment of body segments with respect to gravity. The nature of multisensory interactions has been the subject of a plethora of studies. From the conceptual viewpoint, we will consider below the three myths of postural regulation that have been rather influential in many experimental studies and mathematical models of human posture control: (1) the posture control system is linear, (2) posture control is determined by reflexes, and (3) posture control is equilibrium control.

### Non-linear properties of the posture control system

Small movements accompany the maintenance of any posture. Typically, unless human posture is unstable, body segment oscillations do not exceed 1–2° of joint movements and the CoP oscillations are about 1–2 cm. The fact that postural oscillations are small supports the assumption that the system is linear within a limited range of movements and, therefore, linear computational models and analyses can be applied (Winter et al., 2003; Mergner, 2007; Kiemel et al., 2008; Assländer and Peterka, 2014). While this assumption is valid to some extent and many studies provided very important information about postural strategies and the contribution of different sensory inputs to balance control, one should have in mind that there is also substantial non-linearity in the postural control system, which is often overlooked.

First of all, some non-linearity exists already at the level of muscles, since their resistance to small angular perturbations (~1°, corresponding to about 1% changes in the muscle fiber length, so-called “*short range stiffness*,” Rack and Westbury, 1974) is much higher than the resistance to larger perturbations. Even though the short range stiffness of active calf muscles might not be sufficient to fully compensate the body sway during quiet standing (Morasso and Schieppati, 1999; Loram et al., 2007), its contribution is definitely essential (Gurfinkel et al., 1995). Thixotropy of skeletal muscles (Gurfinkel et al., 1989a) further contributes to the time-dependent augmentation of muscle stiffness for long-sustained postural movements. Indeed, the short-range stiffness component is smaller during periods of high postural sway. Thus, there is a significant reduction (up to 43%) in intrinsic ankle stiffness during conditions of increased baseline sway (Sakanaka et al., 2016), indicating remarkable effects of sway history. Intrafusal fibers of muscle spindles also show thixotropic behavior, implying history-related proprioceptive gain (Proske et al., 1993). In sum, ignoring the non-linear dependence of ankle stiffness on sway size may lead to serious misinterpretation of the results of experiments that use mechanical perturbations or sensory manipulations such as eye closure, movable or unstable support surfaces, sway-referencing, etc. (Loram et al., 2007).

Second, since postural oscillations are small, there are considerable non-linear redistributions of internal displacements of muscle fibers, tendons and soft tissues inside the body. For instance, due to the compliant Achilles tendons, there is paradoxical shortening of soleus and gastrocnemius muscles when the body sways forward and lengthening when the body returns, leaving uncertain the postural role of the numerous calf muscle spindles in the detection of body sway (Loram et al., 2004). Furthermore, the control of equilibrium and internal displacements (of muscle fibers, ligaments and soft tissues) are not restricted to distal joints. For instance, postural disturbances may result from respiratory movements of the thorax and abdomen and should be compensated by movement of the lower limbs and pelvis (Hodges et al., 2002). Moreover, postural stability requires constant activity of axial muscles to stabilize the trunk (and head) and to compensate for movements of the distal parts of the body, if necessary. Finally, the human foot is subjected to considerable deformations during quiet standing due to small CoM displacements and deformations of the soft tissues and the arch of the foot. It is worth stressing that ~0.5 mm vertical oscillations of the calcaneus (and forefoot) observed during quiet standing in healthy adult individuals (Gurfinkel et al., 1994) produce about 0.5° of body tilt (~0.7 cm CoP displacements) even in the absence of ankle joint displacements. In young children, these deformations and their influences on posture control are expected to be even larger since a child's foot goes through significant developmental changes in shape and soft tissues of the foot sole (e.g., the presence of a fat pad underneath the foot plantar surface in infants), once the child starts to stand and walk. Moreover, development of the bony structure of the longitudinal arch only starts ~1 year after birth and continues up to the age of 5 years (Straus, 1926; Maier, 1961). Postural activity of numerous intrinsic foot muscles (that is typically not recorded in postural studies) further contributes to human foot plasticity. There are also large individual differences in foot deformations. These deformations yield large errors in the measured changes of the ankle joint angle, as well as even minute local foot deformations elicit noticeable directional postural responses (Gurfinkel et al., 1994; Wright et al., 2012). However, many postural studies tend to focus on the simple hinge action of the ankle joint (Gatev et al., 1999; Masani et al., 2003; Winter et al., 2003; Mergner, 2007).

The processing of the CoP oscillations imply a certain degree of non-linearity. Upright postural control during quiet standing has often been investigated by quantifying spontaneous postural sway in the displacement, velocity and frequency domains. Nevertheless, the analysis and interpretation of the findings should be carried out carefully since the data processing technique may affect the structure of CoP variability (Rhea et al., 2015). In addition, the similar amplitudes of the CoP oscillations in different animals (Figure 1) raise an important point about their normalization to the body height, body mass and the size of the base of support. Could it reflect an evolutionary adopted sensory threshold for the control of postural sway? Indeed, despite differences in body size, the proprioceptive thresholds (for muscle spindles, joint and load receptors), nerve conduction velocities and the types of muscle fibers are similar for terrestrial mammals, suggesting that a simple size-scaling cannot be applied when comparing sensorimotor control across species (More et al., 2010). Whatever the exact mechanism for comparable CoP oscillations (Figure 1), both mechanical and neural factors are likely to contribute (Gatev et al., 1999; Masani et al., 2003; Winter et al., 2003; Di Giulio et al., 2009; Simoneau and Teasdale, 2015). These considerations are also important for the developmental studies. For example, CoP oscillations are similar or larger (but never smaller) in young children with respect to adult humans notwithstanding over the 2-fold difference in body height (Oba et al., 2015). To some extent, they could be accounted for by the development of postural stability in children. However, we do not know the quantum of CoP oscillations attributable to instability and which proportion may be adjusted due to “unknown” normalization procedure. In other words, we are uncertain about whether and how the CoP amplitude should be normalized to the body height for the same animal at different developmental ages.

There are also other non-linear properties of the sensorimotor system, including thresholds (e.g., for vestibular stimulation), time delays of proprioceptive feedback and neuromuscular delays of force production. The nonlinear geometry of musculo-skeletal connections (e.g., the dependence of the moment arm of muscles upon joint angle) contributes to non-linear properties of the sensorimotor system, though this type of non-linearity is more noticeable during relatively large movements or postural perturbations or when changing the postural set. We will not review here numerous postural models and refer to other articles related to nonlinear control strategy, including burst-like muscle activations, observed especially during unstable conditions. It has even been suggested that intermittent open loop control may be an appropriate solution to deal with feedback time delays, motor noise and computational-muscular economy (Loram et al., 2011). The shift of paradigms in future experimental or modeling studies may be related to the development of non-linear approaches (Loram et al., 2011; Nomura et al., 2013; Funato et al., 2016), although complexity of the model may come at the cost of understanding. These limitations force a necessary trade-off between the usage of linear approaches and more complex postural models. Nevertheless, even if we for simplicity apply linear computations (for instance, Kiemel et al., 2008; Assländer and Peterka, 2014), we need to keep in mind considerable non-linearity in the neuromuscular control of posture.

### Posture control as a summation of postural reflexes

Early postural studies made an emphasis on the reflex nature of postural mechanisms and provided various important examples of static postural reactions (Magnus, 1924; Roberts, 1978). The idea of stretch reflexes, sensory (proprioceptive, visual and vestibular) feedback and its impairment in various forms of pathology of the spinal cord, brainstem and cerebellum, in conjunction with the later developed concept of servoregulation, has been influential in the assessment and modeling of human posture control.

On the other hand, it has been realized that the notion of postural reflexes is rather limited to account for the actual complexity of posture control, which includes anticipatory or feedforward adjustments, context-dependent sensorimotor (or “reflex”) modulations, postural body scheme, and integration of posture and movements (Massion, 1994). A noteworthy illustration of the postural body schema is the modulation of automatic postural reactions (e.g., in response to galvanic vestibular stimulation, muscle vibration, or postural perturbation) according to an illusionary rather than real position of the head or body segments (Gurfinkel, 1994). There are several techniques to artificially induce a dissociation between real and perceived body configuration: by eliciting proprioceptive illusions, by using the phenomenon of “return” of subjective head position to the neutral position after its prolonged turning, or by hypnotic suggestion. All these techniques show similar effects on spatially-oriented postural responses to sensory stimulation. Changes in the gaze direction may also modulate postural responses (Ivanenko et al., 1999), consistent with supraspinal or cognitive influences on posture control, likely because the gaze represents an important reference frame for the internal model of spatial orientation. Thus, the fact that automatic postural reactions are accomplished in accordance with internal representation of body scheme (Popov et al., 1986; Smetanin et al., 1988; Gurfinkel, 1994) indicates that it does not only serve for conscious perception of position but it is also the basis for planning and implementing motor activity. The control of balance during both standing and movements depends on a complex interaction of physiological mechanisms, high level processing of sensory information in accordance with the postural body scheme and on the individual's expectations, goals, cognitive factors and prior experience. The body scheme elements exist already at the level of the spinal cord and contribute to the processing of sensory input and postural responses (Fukson et al., 1980; Windhorst, 1996; Poppele and Bosco, 2003). The notion of body schema has received attention in a large context of contemporary motor control to understand adaptability of reflex modulation, a range of processes such as state estimation, prediction, learning, and to bridge the gap between cognitive and motor functions (Gurfinkel, 1994; Maravita and Iriki, 2004; Windhorst, 2007; Pearson and Gramlich, 2010; Ivanenko et al., 2011; MacPherson and Horak, 2012; Herzfeld and Shadmehr, 2014).

In sum, postural control is no longer considered one system or a given set of equilibrium reflexes but rather a motor skill (Horak and Macpherson, 1995). Many studies are focusing on quantifying the reflex gain of specific neural pathways, such as the Hoffman reflex, local stretch reflexes in individual joints, motor evoked potentials, etc. or applying a specific balance test. They provide knowledge about excitability of these pathways in specific conditions. However, the view that a few pathways or centers in the brain are responsible for posture control is quite limiting in our abilities to assess risks of falling and to improve balance. In addition, high-level cortical involvement increases as postural challenges or demands for reactive control increase (Ouchi et al., 1999; Solopova et al., 2003; Varghese et al., 2015). From the diagnostic and rehabilitation viewpoints, “*many systems need to be evaluated to understand what is wrong with a person's balance”* (Horak, 2006).

### Posture control and equilibrium control

It is typically stated in many articles on posture control that sensory information from somatosensory, vestibular and visual systems are integrated to provide equilibrium maintenance (Fitzpatrick and McCloskey, 1994; Blouin et al., 2007; Mergner, 2007; Assländer and Peterka, 2014; Chiba et al., 2016). Accordingly, a consistent bulk of research focused on postural equilibrium investigates how sensory inputs are reweighted or how neural strategies change in different situations to control balance and postural reactions to perturbations (Nashner, 1976; Ivanenko et al., 1997; Jeka et al., 2004; Schweigart and Mergner, 2008; Nardone and Schieppati, 2010; Simoneau and Teasdale, 2015; Balestrucci et al., 2017). However, the system of posture control must deal with the two tasks simultaneously, one sets a distribution of tonic muscle activity (“posture”) and the other is assigned to compensate for internal or external perturbations (“equilibrium”). Are these two tasks equivalent?

To start with, the control of movement and maintaining a fixed limb posture following movement (holding the body part at its destination) involve distinct neural circuits in the brain stem, cerebellum, motor cortex, hippocampus, etc. (Shadmehr, 2017). For instance, many neurons in the primary motor cortex that express load-related activity are exclusively involved during either posture only or movement only, i.e., they respond differently to transient and continuous loads applied during posture (Kurtzer et al., 2005; Herter et al., 2009). It was suggested that the necessity of having a “hold circuit” may have arisen from the need to maintain a constant “sensory state,” while circuits that are responsible for moving the body part change its sensory state. Since the two tasks (movement and holding still) are inherently interrelated, there is also overlapping and interaction between these circuits. Nevertheless, they differ significantly. Neurophysiological data across different modalities regarding the control of gaze, head movements, arm movement, posture and locomotion indicate that distinct interneurons and motoneurons exhibit bursts of activity during transient movements vs. a sustained level of discharge during posture maintenance (Shadmehr, 2017). Accordingly, a similar concept can be applied to the control of phasic and tonic postural muscle activity. As far as it concerns postural tone, it originates from several supraspinal centers, including the reticular formation, vestibular nuclei, cerebellum, and mesodiencephalic nuclei (Hess, 1954). These brain regions can exhibit sustained long-lasting activity providing a prolonged excitation and inhibition of executive motor systems. In addition, there are also specialized pathways to the spinal cord (Kuypers, 1964; Szokol and Perreault, 2009; Deliagina et al., 2014) and specialized activation of the trunk musculature during various postural and motor tasks (Urquhart et al., 2005; Falgairolle et al., 2006, 2013; Tsao et al., 2011; Beliez et al., 2015). For instance, descending pathways to the axial musculature (that links all parts of the body together and provides axial muscle tone and trunk stabilization) via somatic descending brain stem and monoaminergic pathways are distinct from the descending tracts to limb motoneurons (Kuypers, 1964; Szokol et al., 2008; Sivertsen et al., 2014).

Slow and fast processes in the central nervous system are also often linked to the control of muscle tone and phasic muscle activity. For instance, various postural aftereffects are associated with slow changes in the tonic muscle activity (Gurfinkel et al., 1995; Kluzik et al., 2005; Bove et al., 2009; Wright, 2011). In some conditions, posture-related and equilibrium-related control can be differentiated with regard to slow and fast components of CoP displacements, respectively. For instance, participants with occluded vision undergoing super slow (<0.1°/s) tilts of the supporting platform, subthreshold for most vestibular and proprioceptive phasic reactions, display very large compensatory phase shifts and delays (tens of seconds). It is worth noting, though, that large slow body movements are superimposed with small irregular oscillations reflecting an ongoing equilibrium control (Gurfinkel et al., 1995). Thus, besides operative control assigned to compensate deviations from a reference position, the system of postural control includes at least one additional level, which elaborates this postural “set” taking into account the energy cost of standing, position of body segments, muscular torques and demands for stability and security. From a functional point of view, this may solve the old *posture-movement paradox* introduced by a famous German scientist, Erich von Holst (1908–1962): how we can move from one posture to another without triggering resistance from posture-stabilizing mechanisms. If one considers posture and equilibrium to be mediated by distinct neural circuits, posture-stabilizing mechanisms may be responsible for the control of equilibrium relative to the superiorly determined postural set.

The basis of habitual sitting or standing human posture is postural tone of skeletal muscles. Phasic activity is often voluntary (though it may be automatic as well) while tonic involuntary activity is less known and much less studied. There are methodological difficulties since the activity in many (e.g., trunk) muscles is rather small. Under narcosis, muscle tone disappears while tonic activity can be observed during sleeping, since there are some active phases accompanied by muscle tonic contractions (Harris, 2005; Peever, 2011; Huon and Guilleminault, 2017). Among important examples of long-lasting involuntary activity are the tonic vibration reflex (Eklund and Hagbarth, 1966) and involuntary postcontraction muscle activity (Salmon, 1914; Kohnstamm, 1915) that have been suggested to represent an amplification of neuromotor processes normally involved in automatic posture maintenance and tonic spinal activity (De Havas et al., 2017; Ivanenko et al., 2017).

An important issue is an evaluation and definition of muscle tone (Gurfinkel et al., 2011), which is traditionally linked to the activity level of muscle. In clinical practice, changes in tonus are typically measured, not tonus *per se*, by the extent of the muscle resistance to stretch. However, muscle length changes may also evoke involuntary shortening reactions (compliant posture behavior) or elicit postural adjustments of other “remote” muscles not being primarily stretched (Andrews et al., 1972; Gurfinkel et al., 1989b). A dynamic “postural frame,” that is inherently incorporated in posture and movement coordination, may account for the resistive or compliant behavior of the body (Cacciatore et al., 2014). In this respect, Bernstein's (1940) interpretation of muscle tone seems more functional, as *the degree of readiness for movement* related to movement as a state is related to an action, or as a precondition is related to an effect. Changes in muscle tone affect movements. The remarkable findings of the British neurologist Martin (1967) provide excellent examples of how disturbances to postural tone in humans affect the ability to perform movements. For instance, the loss of normal posture of the head and trunk can be observed in patients with eyes closure while inability to hold the body up may result in a gradually flexed posture during walking. Furthermore, disturbances of trunk posture, its dynamics and variability during walking may differ for idiopathic and parkinsonian camptocormia, suggesting the involvement of different underlying physiopathological mechanisms (de Sèze et al., 2015). In addition, trunk postural adjustments may also depend on walking conditions, for instance, forward vs. backward walking (Ivanenko et al., 2013). These disturbances are related primarily to automatic rather than voluntary control of posture (Wright et al., 2007; Ivanenko et al., 2013). The level of tonic muscle activity substantially influences postural orientation (Martin, 1967; Kluzik et al., 2005; Wright, 2011) and is inherently incorporated in gait control (Mori, 1989).

In summary, the central nervous system is able to combine mobility with stability and the nature of interactions between posture and movement is a long-standing problem in movement neuroscience. The latter aspect was best described by Sherrington (1906) more than a century ago—“posture follows movement like a shadow.” It even anticipates movement. Tonic muscle activity and posture control require specialized neural circuitry. An appropriate postural tone is an integral part of any movement and disturbances to muscle tone may in turn affect movement performance. In order to understand the control of posture and movements, we need to know better how postural tone is generated and maintained, including its neuromuscular underpinnings.

## Author contributions

All authors listed have made a substantial, direct and intellectual contribution to the work, and approved it for publication.

### Conflict of interest statement

The authors declare that the research was conducted in the absence of any commercial or financial relationships that could be construed as a potential conflict of interest.

## Abbreviations

CoM: centre of body mass
CoP: centre of pressure.
